# Polychromatic Iterative Statistical Material Image Reconstruction for Photon-Counting Computed Tomography

**DOI:** 10.1155/2016/5871604

**Published:** 2016-03-17

**Authors:** Thomas Weidinger, Thorsten M. Buzug, Thomas Flohr, Steffen Kappler, Karl Stierstorfer

**Affiliations:** ^1^Institute of Medical Engineering, University of Lübeck, Ratzeburger Allee 160, 23538 Lübeck, Germany; ^2^Siemens AG, Healthcare Sector, Imaging & Therapy Division, Siemensstraße 1, 91301 Forchheim, Germany

## Abstract

This work proposes a dedicated statistical algorithm to perform a direct reconstruction of material-decomposed images from data acquired with photon-counting detectors (PCDs) in computed tomography. It is based on local approximations (surrogates) of the negative logarithmic Poisson probability function. Exploiting the convexity of this function allows for parallel updates of all image pixels. Parallel updates can compensate for the rather slow convergence that is intrinsic to statistical algorithms. We investigate the accuracy of the algorithm for ideal photon-counting detectors. Complementarily, we apply the algorithm to simulation data of a realistic PCD with its spectral resolution limited by K-escape, charge sharing, and pulse-pileup. For data from both an ideal and realistic PCD, the proposed algorithm is able to correct beam-hardening artifacts and quantitatively determine the material fractions of the chosen basis materials. Via regularization we were able to achieve a reduction of image noise for the realistic PCD that is up to 90% lower compared to material images form a linear, image-based material decomposition using FBP images. Additionally, we find a dependence of the algorithms convergence speed on the threshold selection within the PCD.

## 1. Introduction

The development of dual-energy (DE) CT scanners paved the road for new clinical applications, taking advantage of the energy dependence of X-ray attenuation in matter, a fact that formerly had been considered a drawback. This technology allows for exposing patients to two distinct spectra, acquiring information about the material composition of the interior of the body. Besides scanners with two X-ray sources and detectors [1] there also exist approaches that, for instance, apply a fast kVp switching technique [2] or dual-layer detectors [3, 4] to acquire spectrally resolved scan data. All those approaches have in common that they are hardly extendable to produce more than two spectrally well separated data sets. Additionally, spectrally resolved data from dual-source dual-energy and fast kVp switching devices do not match exactly due to a time shift between measured sinograms or projections, respectively, making a correct statistical treatment of the problem in image reconstruction difficult.

The continuous improvement of photon-counting detectors (PCDs) over the past few years promises a remedy for this limitation and holds new possibilities for multienergy imaging. Recent prototype-detectors already provide two to four [5, 6] discriminator thresholds. This allows the acquisition of two to four sinograms in a single scan, each sensitive to a different part of the applied tube spectrum. Apart from energy-resolved scan data, photon-counting technology can provide further benefits, like reduced image noise [7] and enhanced contrast [8, 9].

However, there are also issues with the new detector type concerning its capability of high X-ray fluxes which may occur in clinical CT examinations [10]. The consequence of high photon flux is the occurrence of pulse pileup. The severeness of pileup correlates with the detector pixel pitch. Additionally, K-escape events and crosstalk between adjacent pixels lead to a degradation in spectral resolution. Their influence is inversely correlated with the detector pixel pitch. So the challenge is to find a compromise between spectral sensitivity and high flux-rate capability to ensure the clinical usability of photon-counting detectors.

In established dual-energy applications, the spectral information is used, for instance, to classify different body materials, identify high contrast agent concentrations that indicate pathological tissue, or support an early diagnosis of gout by helping to distinguish urate from small calcifications [11, 12]. Other applications utilize the spectral information to remove osseous structures in the patient image, revealing previously covered structures [13].

Usually, a linear material decomposition is performed subsequently to image reconstruction, although raw data based approaches have already been investigated [14]. As a consequence of the material decomposition process, image noise is increased significantly, especially if more than two materials are to be separated [6].

Statistical reconstruction methods offer the opportunity to achieve images with reduced noise compared to convenient filtered back-projection (FBP) reconstructions by including mature regularization techniques [15]. Statistical methods account not for the physical effects governed by the absorption characteristics of matter only. They also respect the Poisson nature of the emission and absorption processes in X-ray tubes and detectors, respectively. The correct statistical treatment becomes relevant in PCDs with high spectral resolution. Impinging photons are distributed into energy bins according to the energy assigned to them in the detection process. Therefore, an increase in energy resolution implies smaller energy bins with reduced photon statistics for a constant number of impinging photons and constant spatial sampling.

Statistical algorithms converge to the most likely image that best fits the measured sinogram data, according to the underlying statistical model. The canonical measure for how well image and measured data agree is given by the logarithmic likelihood function (log-likelihood), which must be maximized to find the best suiting image. Since no analytic solution exists for the maximum of the Poisson log-likelihood function it must be approximated by numerical methods. Some years ago, a new class of statistical, polychromatic algorithms has been introduced [16, 17] that exploit the convexity of the* negative* log-likelihood by minimizing surrogate functions instead. Surrogate functions are successive local approximations of the negative log-likelihood function. With surrogates it is possible to formulate the optimization problem in a parallel manner, which allows quick computation on graphic processing units (GPUs). Under certain circumstances [18] the monotonic convergence of the algorithm is provable. The algorithm's polychromatic nature also implies a beam-hardening correction (BHC) for the chosen basis materials (cf.  Section 2.1.3). With quite strong empirical assumptions the algorithm can be applied to spectrally not resolved data (e.g., to sinogram data taken with a convenient CT system with an energy integrating detector) with good results [19, 20].

In our approach we drop these assumptions and reconstruct the material fractions directly from energy-resolved sinogram data. Compared to [21] our algorithm allows a simultaneous and parallel update of all material images and considers the detector response function of a photon-counting detector. The algorithm is a priori not limited to two spectrally well separated data sets but scales with the number of energy-resolved data sets. As the number of thresholds in future PCDs might increase an extension of the material separation to the respective number of basis materials will be possible, as long as these materials are distinguishable by means of energy-resolved CT (cf.  Section 2.1.3). Our intention is to evaluate the accuracy of the reconstructed material fractions and determine the anticipated gain in image quality due to image noise reduction from a correct statistical treatment of the problem.

In Section 2 we introduce the mathematical background and describe the setup of this study.  Section 3 presents the results that are discussed subsequently in Section 4.

## 2. Materials and Methods

### 2.1. Theoretical Background

#### 2.1.1. Statistical Model: Poisson Random Variables

The creation of X-ray photons and their absorption and registration in a counting detector pixel are statistical processes that can be described by a Poisson random variable ${𝒫}_{i}\left(N_{i}|{\bar{N}}_{i}\right)$ . In computed tomography the joint probability for the measurement of a complete sinogram is thus the product of the individual random variables of all sinogram pixels

(1) $$\begin{array}{c} P\left(\;N\;|\;\bar{N}\;\right)=\overset{M}{\underset{i=1}{\prod }}P_{i}\left(\;N_{i}\;|\;{\bar{N}}_{i}\;\right)=\overset{M}{\underset{i=1}{\prod }}\frac{{\bar{N}}_{i}^{N_{i}}}{N_{i}!}e^{-{\bar{N}}_{i}}. \end{array}$$

Here *i* indexes all *M* projection values that constitute the complete sinogram. In photon-counting CT *N*_*i*_ is the number of pulses registered in a single detector pixel and ${\bar{N}}_{i}$ the corresponding expectation value. The negative logarithm of $𝒫\left(N‍|\bar{N}\right)$ yields the canonical negative log-likelihood function which can be used as a measure for how well taken data **N** agree with their expectation values $\bar{N}$

(2) $$\begin{array}{c} -L\left(\;\bar{N}\;\right)=-\log\left(\;P\left(\;N\;|\;\bar{N}\;\right)\;\right)=-\overset{M}{\underset{i=1}{\sum }}N_{i}\log{\bar{N}}_{i}+{\bar{N}}_{i}. \end{array}$$

We use bold letters to indicate vector quantities. Terms that are constant with respect to ${\bar{N}}_{i}$ have been omitted, since the goal of statistical reconstruction is to find the expectation values ${\bar{N}}_{i}$ that best fit the measured data, that is, the minimum of $-L(\bar{N})$ , which is not altered by terms constant with respect to ${\bar{N}}_{i}$ .

In multienergy imaging one can formulate separate log-likelihood functions $L^{b}(N|\bar{N})$ for all sinograms, each associated with one of the *B* energy bins provided by a PCD. If all random variables are statistically independent, which we assume here, an appropriate objective function for minimization is the sum over all *B* negative log-likelihood functions

(3) −LN¯−∑b=1BLbN¯=∑b=1B ∑i=1M−Niblog⁡N¯ib+N¯ib≡∑b=1B ∑i=1MhibN¯ib.

The statistical independence holds true only in the case of an ideal photon-counting detector. In realistic PCDs dependencies between energy bins are introduced by K-escape, pixel crosstalk, electronic noise, and pulse pileup. Our proposed algorithm accounts for correlations introduced by K-escape, pixel crosstalk, and electronic noise by considering the detectors response function, whereas pulse pileup is corrected in a preprocessing step directly on the sinogram raw data.

In general, a minimization of the log-likelihood function alone leads to very noisy images. To compensate for this, a regularization term (prior) *R* is added to the objective function. It incorporates a priori knowledge about the material images *f*_*j*_^*k*^ that are to be reconstructed. Convenient priors suppress small differences between neighboring pixels but preserve contrast edges. They usually take the form

(4) $$\begin{array}{c} R^{k}\left(\;f^{k}\;\right)=\overset{P}{\underset{j=1}{\sum }}\;\underset{l\in N_{j}}{\sum }\psi^{k}\left(\;f_{j}^{k}-f_{l}^{k}\;\right), \end{array}$$

where *P* is the number of image pixels and *l* labels the *𝒩*_*j*_ neighboring pixels of a considered image pixel *j*. The index *k* labels the *K* material images that are to be reconstructed. We require the penalty functions *ψ* to be strictly convex and twice continuously differentiable with respect to *f*_*j*_^*k*^. This requirement is necessary [16] to allow parallelization while assuring convergence of the final algorithm. We implemented a prior suggested by Green [22]. The computational effort for this prior is higher compared to frequently utilized quadratic priors, but since it is a differentiable approximation of the Huber prior [23] it can better preserve contrast edges. The addition of the prior term to the negative log-likelihood function yields

(5) $$\begin{array}{c} \Phi \left(\;f\;\right)=-L\left(\;f\;\right)+\eta R\left(\;f\;\right) \end{array}$$

as new objective function, with the scalar product

(6) $$\begin{array}{c} \eta R\left(\;f\;\right)=\overset{K}{\underset{k=1}{\sum }}\eta^{k}R^{k}\left(\;f^{k}\;\right). \end{array}$$

The parameter **η** ≡ {*η* ^*k*^} governs the strength of regularization. Moreover, we made use of the fact that the number of pulses *N*_*i*_ registered in a sinogram pixel depends on the fractions **f** of materials constituting the scanned object; that is, *N*_*i*_ = *N*_*i*_(*f*_*j*_^*k*^). The dependence is given by Lambert-Beer's law in (7) and (9) as described below.

#### 2.1.2. Physical Model: Polyenergetic Lambert-Beer's Law

The goal of computed tomography is to reconstruct an attenuation map *μ*_*j*_ describing the interior of a scanned object. The attenuation map cannot be measured directly, but only via projections *N*_*i*_ of the object. The link between the attenuation map *μ*_*j*_ and its projections *N*_*i*_ is given by Lambert-Beer's law. This model correctly reflects the physical behavior for a monoenergetic X-ray source. But in clinical practice X-rays are created by X-ray tubes which emit quanta distributed over a continuous spectrum *𝒮*(*E*) of energies. Image reconstructions based on Lambert-Beer's law, like filtered back-projection (FBP), neglect the polychrome nature of the X-rays and can therefore lead to serious beam-hardening artifacts that degrade image quality. The artifacts need to be corrected, which usually happens in a preprocessing step. To avoid their generation already in the reconstruction process, it is necessary to know the materials composing the object and integrate Lambert-Beer's law for polyenergetic X-rays (cf. [15])

(7) N¯ib∫Emin,bEmax,bNi,0SbEexp⁡−∑j=1PaijμjEdE=∫Ni,0bEexp⁡−∑j=1PaijμjEdE,

into the reconstruction algorithm. In (7) *N*_*i*,0_^*b*^(*E*) is defined as *N*_*i*,0_^*b*^(*E*) ≡ *N*_*i*,0_(*E*)*𝒮* ^*b*^(*E*) and *𝒮* ^*b*^ is the spectrum the *b*th energy bin is sensitive to. For realistic PCDs *𝒮* ^*b*^(*E*) can be calculated from the tube spectrum *𝒮*(*E*) via

(8) $$\begin{array}{c} S^{b}\left(\;E\;\right)=\overset{E_{max}^{\prime b}}{\underset{E_{min}^{\prime b}}{\int }}S\left(\;E\;\right)\Sigma \left(\;E,E^{\prime }\;\right)dE^{\prime }, \end{array}$$

where Σ(*E*, *E*′) is the normalized detector response function. It states the probability of the detector assigning energy *E*′ to a measured photon with true energy *E*. The system matrix *A* ≡ {*a*_*ij*_} governs the contribution of the *j*th image pixel to the *i*th projection, so it implicitly contains the scanner geometry.

#### 2.1.3. Material Composition Model

Neglecting Rayleigh scattering, there are two physical effects relevant for the attenuation in clinical CT examinations: Compton scattering and photoelectric effect. This fact would limit the number of materials separable with energy-resolved CT to *K* = 2. Fortunately, due to the unique spectral lines of each chemical element more than two materials can be separated, if at least *K* − 2 of the *K* materials exhibit one or more individual spectral lines in the range of the applied X-ray spectrum and all of them are mutually exclusive regarding their constituting chemical elements. Under that assumption, we can decompose each material into a linear combination of *K* basis materials with their respective energy-depending attenuation coefficient *μ* ^*k*^(*E*)

(9) $$\begin{array}{c} \mu_{j}\left(\;E\;\right)=\overset{K}{\underset{k=1}{\sum }}f_{j}^{k}\mu^{k}\left(\;E\;\right). \end{array}$$

The number of materials *K* forming the material basis may not exceed the number *B* of available spectrally different data sets to guarantee that the system of equations is not underdetermined. The choice of the material basis must depend on the composition of the scanned object and is crucial for the accuracy of the composition estimation. A suitable choice for clinical applications should include water, since it resembles well the attenuation behavior of the majority of human tissue [24–26]. Additional basis materials should be chosen dependent on the imaging task. Calcium or a suiting mixture of materials could be included if osseous body parts are to be identified. If an injected iodine-based contrast agent is to be identified, which is frequently used in clinical CT examinations to enhance the contrast of cancerous tissue, iodine should be included.

A correct and reliable separation of materials requires a different absorption behavior of each basis material in the range of the applied tube spectrum. If attenuation coefficients of different materials have the same energy dependency in the energy range covered by the spectrum, those materials cannot be distinguished by means of spectrally resolved CT.

#### 2.1.4. Optimization Transfer Principle

**Figure 1:**
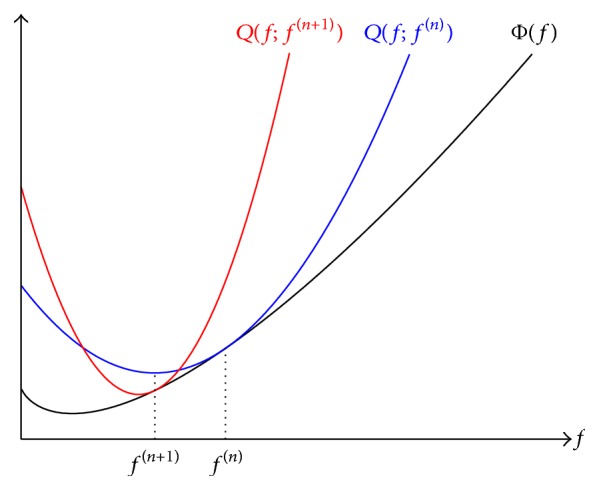
One-dimensional illustration of the optimization transfer principle. *Q*(*f*; *f* ^(*n*)^) is a surrogate function to the objective function Φ(*f*) at iteration step (*n*). Hence, it is tangential to Φ(*f*) at *f* ^(*n*)^. The abscissa of the minimum of *Q*(*f*; *f* ^(*n*)^) is the new estimate *f* ^(*n*+1)^. It in turn marks the position where the next surrogate function *Q*(*f*; *f* ^(*n*+1)^) is constructed, tangential to Φ(*f*).

The proposed algorithm applies the optimization transfer principle introduced by De Pierro [16, 27]. According to this principle, the objective function is locally approximated by surrogate functions that are easier to minimize (cf.  Figure 1). In fact, we will approximate the objective function by parabolic surrogates whose minima are known analytically. A new surrogate function is generated in each iteration step (*n*). The following conditions imposed to a surrogate *Q* are sufficient [18] to guarantee convergence of the algorithm:

(10) Qfn;fn=Φfn,∂Qf;fn∂fjkf=fn=∂Φf∂fjkf=fn,Qf;fn≥Φf.

Equations (10) ensure that each surrogate always lies completely above the objective function Φ(**f**) and is tangential to it at the current iteration step.

### 2.2. Algorithm Derivation

#### 2.2.1. Surrogate Objective Functions

Our derivation of the separable surrogate objective function and the update equation follows the procedure presented in [19]. In a first step, the energy integral in the physical model, (7), is moved out of the log-likelihood term of the objective function (5). Therefore, we define

(11) likE,fk∑j=1PaijfjkμkE,

(12) tiE,f∏k=1KtikE,fk=exp⁡−∑k=1KlikE,fk≡exp⁡−liE,f,

(13) βib,nEN¯ib,nE,fntiE,fn.

With these definitions, the polychromatic Lambert-Beer law (7) can be rewritten as

(14) $$\begin{array}{c} {\bar{N}}_{i}^{b}\left(\;f\;\right)=\int \frac{N_{i,0}^{b}\left(E\right)}{\beta_{i}^{b,\left(n\right)}\left(E,f^{\left(n\right)}\right)}t_{i}\left(\;E,f\;\right)\beta_{i}^{b,\left(n\right)}\left(\;E,f^{\left(n\right)}\;\right)dE. \end{array}$$

Since ${\bar{N}}_{i}^{b}(f)$ is a convex function and it holds that

(15) $$\begin{array}{c} \int \frac{N_{i,0}^{b}\left(E\right)}{\beta_{i}^{b,\left(n\right)}\left(E,f^{\left(n\right)}\right)}dE=1, \end{array}$$

we can replace −*L*(**f**) by the surrogate *Q*_1_(**f**, **f** ^(*n*)^) by applying Jensen's inequality for convex functions

(16) −Lf≤∑b=1B ∑i=1N∫Ni,0bEβib,nE,fn·hibtiE,fβib,nE,fndE≡Q1f,fn.

It can be shown that *Q*_1_(**f**, **f** ^(*n*)^) fulfills conditions (10). Next, we want to approximate *Q*_1_ by another parabolic surrogate whose minimum is known analytically. To this end, expand *Q*_1_ into a Taylor series up to the second order about the current line integrals {*l*_*i*_^*k*,(*n*)^}

(17) hibE,li≤qib,nE,lik

(18) =hibE,lin+∑k=1K∂hibE,li∂liklik=lik,nlik−lik,n+12∑k=1K ∑m=1KCibkm,nlik−lik,nlim−lim,n.

We end up with the new surrogate function

(19) $$\begin{array}{c} Q_{2}\left(\;f,f^{\left(n\right)}\;\right)=\overset{B}{\underset{b=1}{\sum }}\;\overset{N}{\underset{i=1}{\sum }}\int \frac{N_{i,0}^{b}\left(E\right)}{\beta_{i}^{b,\left(n\right)}}q_{i}^{b,\left(n\right)}\left(\;E,l_{i}^{k}\;\right)dE. \end{array}$$

The curvature *C*_*i*_^*bkm*,(*n*)^ in (17) must be chosen such that the optimization transfer principle (10) is satisfied. Practically, we will use the Hessian of *h*_*i*_^*b*^(*E*, *l*_*i*_) instead. With this choice the monotonicity of the algorithm cannot be guaranteed mathematically anymore. If we forced the line integrals *l*_*i*_^*k*^ to be positive, there would exist curvatures that provably ensures monotonic convergence [18]. However, this constraint might affect the capability of the algorithm to model materials not incorporated in the material basis. Materials not part of the basis are represented as a linear combination of the basis materials (see (9)) which also requires negative coefficients *f*_*j*_^*k*^. A weaker form of the positivity constraint merely postulates that ∑_*k*=1_^*K*^ *f*_*j*_^*k*^ ≥ 0 would be compatible with the model. Whether this constraint still guarantees monotonic convergence and the existence of an optimal (in the sense of convergence rate, cf. [18]) curvature remains an open question.

Finally, we replace *Q*_2_ by a surrogate that allows independent updates of all image pixels, enabling parallel computation. To this end, we rewrite the *k*th line integral as a convex combination by transforming

(20) likE,fk=∑j=1PaijfjkμkE=∑j=1PαijaijμkEαijfjk−fjk,n+liknE=∑j=1PαijλijkE,fjk,

with

(21) αij=aij∑j=1Paij,∑j=1Pαij=1,

(22) liknE=∑j=1PaijμkEfjk,n.

Applying once again Jensen's inequality, we pull the sum over the image pixels *j* out of *q*_*i*_^*b*,(*n*)^(*E*, ∑_*j*=1_^*P*^ *α*_*ij*_ *λ*_*ij*_^*k*^(*E*, *f*_*j*_^*k*^)) which yields our final surrogate function *Q*_3_(**f**, **f** ^(*n*)^)

(23) Q3f,fn=∑b=1B ∑i=1N ∑j=1P∫Ni,0bEβib,nαij·qib,nE,λijkfjkdE.

For the deduction of the separable surrogate *S*(**f**, **f** ^(*n*)^) of the regularization term we followed De Pierro [16] and ended up with
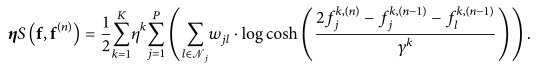
(24)


#### 2.2.2. Minimization Method

To minimize the surrogate objective function *Q*_3_ + **η** *S* we apply the Newton-Raphson method:

(25) fn+1=fn−∇Q3f,fn+Sf,fn·HQ3+HηS−1f=fn,

H_*Q*_3__ and H_**η***S*_ are the Hessian matrices, that is, the second derivatives of *Q*_3_ and **η** *S*, respectively. Hence, the (*n* + 1)st iteration step for the *k*th material image does only depend on the measured data of the *b*th energy bin and the material images previously calculated in the *n*th iteration step. This permits parallel updates of all *K* material images.

Evaluating the gradient of *Q*_3_ at the current iteration **f** ^(*n*)^ yields

(26) $$\begin{array}{c} {\left.\;\frac{\partial Q_{3}\left(f,f^{\left(n\right)}\right)}{\partial f_{j}^{k}}\;\right|}_{f=f^{\left(n\right)}}=\overset{B}{\underset{b=1}{\sum }}\;\overset{N}{\underset{i=1}{\sum }}\left(\;1-\frac{N_{i}^{b}}{{\bar{N}}_{i}^{b,\left(n\right)}}\;\right)\frac{\partial {\bar{N}}_{i}^{b,\left(n\right)}}{\partial f_{j}^{k}}. \end{array}$$

For the elements of the second derivative of *Q*_3_ one gets

(27) ∂2Q3∂fjk∂fjm=∑b=1B ∑i=1N∫Ni,0bEβib,nαijCibkm,n∂λijk∂fjk∂λijm∂fjm=∑b=1B ∑i=1N∫Ni,0bEβib,nαijaij2μkEμmECibkm,ndE.

Using the Hessian ∂^2^ *h*_*i*_^*b*^(*E*, *l*_*i*_)/∂*l*_*i*_^*k*^∂*l*_*i*_^*m*^ of *h*_*i*_^*b*^(*E*, *l*_*i*_) instead of a curvature *C*_*i*_^*bkm*,(*n*)^ that satisfies the conditions of the optimization transfer principle we get

(28) ∂2Q3∂fjk∂fjm∑b=1B ∑i=1N∫Ni,0bEβib,nαijaij2μkEμmENibdE=∑b=1B ∑i=1NaijNibN¯ib∑j=1Paij·∫Ni,0bEμkEμmEexp⁡−lindE.

In the last step *α*_*ij*_ and *β*_*i*_^*b*,(*n*)^ were replaced by their respective definitions (21) and (13). The derivatives of the surrogates *S*(**f**, **f** ^(*n*)^) of the regularization function are

(29) ∂ηS∂fjk=ηk∑l∈Njwjlγk·tanh⁡2fjk,n−fjk,n−1−flk,n−1γk,∂2ηS∂fjk∂fjm=δkmηk∑l∈Nj2wjlγk2·1−tanh2⁡2fjk,n−fjk,n−1−flk,n−1γk,

with the Kronecker symbol *δ*_*km*_.

#### 2.2.3. Summary of the Algorithm

In the following we give a brief step by step overview over the algorithm:

(1) First, one has to compute the line integrals *l*_*i*_^*k*,(*n*)^(*E*, **f** ^*k*,(*n*)^) of the material images by forward-projecting them; see (11). Subsequently, determine *t*_*i*_(*E*, **f** ^*k*,(*n*)^) as given by (12). For an adequate initialization of the algorithm provide the initial material images from material separated FBP images reconstructed from the raw data.
(2) Next, determine the *N*_*i*_^*b*,(*n*)^ by multiplying *t*_*i*_(*E*, **f** ^*k*,(*n*)^) with the mean number of photons *N*_*i*,0_ emitted by the tube and the respective spectrum *𝒮* ^*b*^ the *b*th energy bin is sensitive to. Finally, discretize the integral in (7) an carry out the summation. The spectra *𝒮* ^*b*^ can be calculated from the detector response function as stated by (8).
(3) Additionally, calculate
  (30) $$\begin{array}{c} \int N_{i,0}^{b}\left(\;E\;\right)\mu^{k}\left(\;E\;\right)\exp\left(\;-\overset{P}{\underset{j=1}{\sum }}a_{ij}\mu_{j}\left(\;E\;\right)\;\right)dE, \end{array}$$
 which equates to $\left(1/a_{ij}\right)\left(\partial {\bar{N}}_{i}^{b,(n)}/\partial f_{j}^{k}\right)$ .
(4) Finally, one can determine ∇*Q*_3_(**f**, **f** ^(*n*)^) following (26).
(5) The Hesse-matrix ∂^2^ *Q*_3_/∂*f*_*j*_^*k*^∂*f*_*j*_^*m*^ can be achieved in a similar fashion, using (28). It should be mentioned that (∑_*j*=1_^*P*^ *a*_*ij*_) in (28) is a simple forward-projection of an image containing all ones, so it can be precomputed.
(6) Multiplying the inverted Hesse-matrix with the previously calculated gradient and subtracting the result from the current material images **f** ^(*n*)^ yield the updated material images **f** ^(*n*+1)^.

### 2.3. System Setup

#### 2.3.1. Accuracy Analysis with Ideal PCD Data

**Figure 2:**
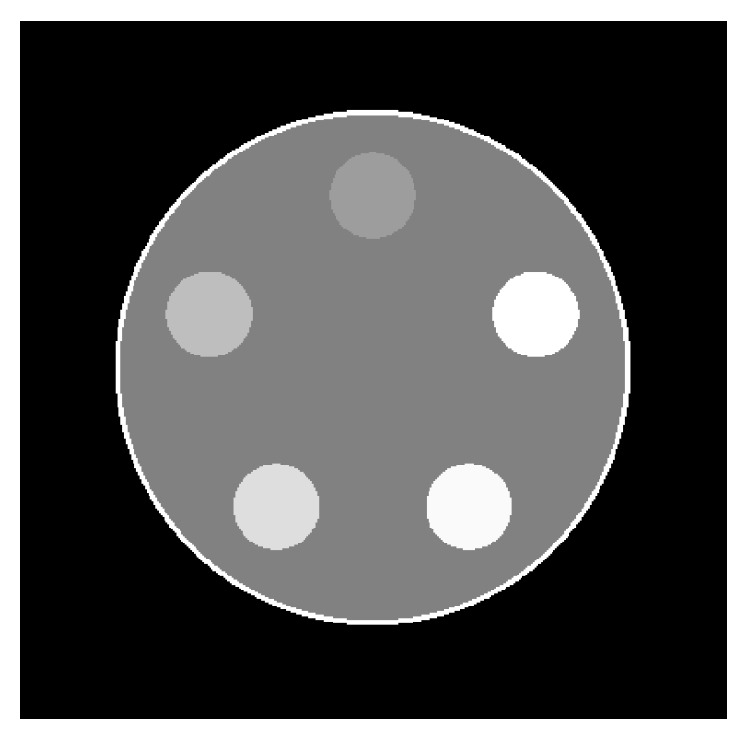
Normalized scaled densities of the modeled 30 cm diameter water phantom containing small cylinders of various concentrations of iodine with windowing of *C* = 0 and *W* = 100.

We started evaluating the accuracy of the algorithm by reconstructing *K* = 2 material images from ideal simulation data of a water filled acrylic glass cylinder, generated with the DRASIM software [28]. In this context, ideal means that the detector perfectly separates the irradiating tube spectrum into *B* disjoint energy bins. Effects like K-escape, charge sharing, electronic noise, and pulse pileup do not occur. For the evaluation of the algorithm we choose the number of energy thresholds and by that the number of energy bins to  *B* = 2, matching the number *K* of material images to be reconstructed. The cylinder phantom has a diameter of 30 cm, containing five small cylinders with various concentrations of iodine. The iodine concentration increases from *f* ^iodine^ = 0.00243 counterclockwise to *f* ^iodine^ = 0.01215 in equidistant steps, with the upper contrast cylinder (cf.  Figure 2), having the lowest concentration.

The scans were simulated in fan-beam geometry with a field of view (FOV) of 52 cm. The isocenter to detector distance was *r*_CD_ = 49 cm and the focus to isocenter distance *r*_FC_ = 60 cm. The virtual detector features 4 rows with 3680 quadratic subpixels, each with a pitch of 250 *μ*m. Data simulated with that geometry are fused to macropixels previous to further processing. A macropixel contains the summed counts of 4 × 4 small pixels. Each fifth column of small pixels is excluded to account for the covering due to a collimator grating.

Over an angular range of 540° 1664 projections were taken, exposing the phantom to a 140 kVp tube spectrum. The additional angular range of 180° in addition to a full cycle is required by the employed rebinning algorithm. Rebinning converts the sinogram data from fan-beam to parallel-beam geometry prior to reconstruction. In doing so, the detector quarter-shift is used to double the sampling.

The tube was driven with a current of 100 mA. The emitted spectrum, irradiated onto the cylinder phantom, was prefiltered with 0.9 mm of titanium and 3.5 mm aluminum. We did not account for a bowtie filter in the simulations, albeit it could be respected in the detector response function (8) via an additional factor that depends on the sinogram pixel index *i*. The simulated detector has two counter thresholds, providing two spectrally separated data sets.

We chose iodine and water as basis materials and initialized the algorithm with the ground truth, that is, the correct material images *f*_*j*_^*k*^. Since acrylic glass is not part of the material basis, the borders of the cylinder phantom will be represented as a linear combination of iodine and water.

**Figure 3:**
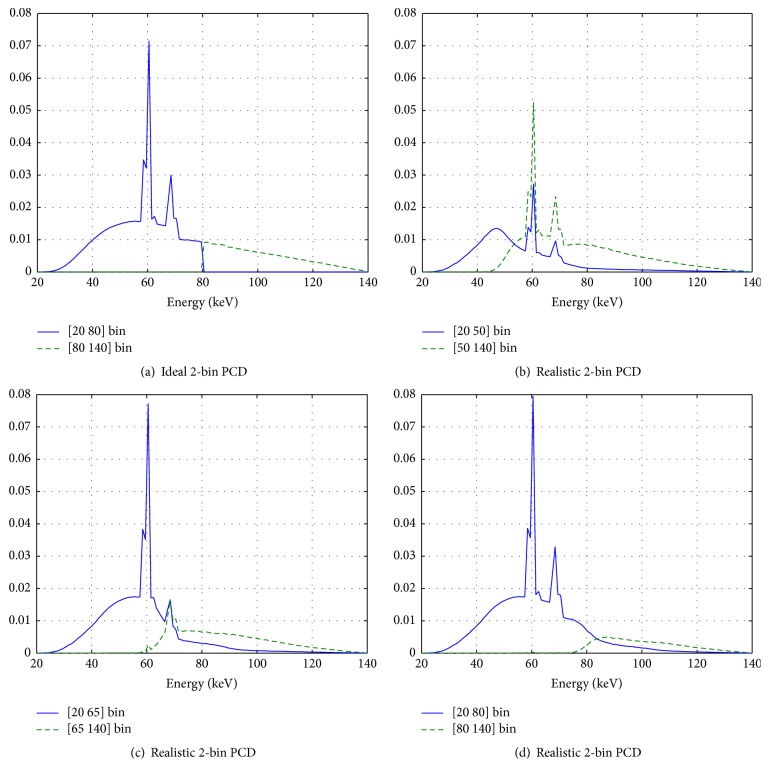
Normalized tube spectrum, prefiltered with 0.9 mm Ti and 3.5 mm Al. Shown are the sensitive ranges of the low bin (blue, solid line) and the high energy bin (green, dashed line) for the ideal (a) and the realistic ((b)–(d)) two-bin PCD, respectively. The energy resolution in the plots is 1 keV.

The energy resolution of the algorithm was set to 5 keV. This determines the precision of approximation of the energy integral in (7). The bin-spectra *𝒮* ^*b*^(*E*) are provided with the same resolution. Bin-spectra are the parts of the tube spectrum the energy bins of a PCD are sensitive to (see Figure 3(a)). The mass attenuation coefficients *m* ^*k*^(*E*) and the material densities *ρ* ^*k*^ for the calculation of attenuation values *μ* ^*k*^(*E*) = *m* ^*k*^(*E*)*ρ* ^*k*^ were taken from the EPDL library [26] and Kuchling [29], respectively. To evaluate the mass attenuation coefficients at the sample points of the bin-spectra the mass attenuation coefficients from the EPDL library were interpolated. The interpolation was conducted piecewise if the considered material possesses one or more absorption edges within the energy range of the bin-spectra.

#### 2.3.2. Dependence of Convergence Speed on Energy Bin Selection

Real photon-counting detectors are not able to separate the registered photons into perfectly disjoint energy bins. This is mainly a consequence of signal sharing between neighboring pixels. Together with pulse pileup, it leads to a considerable overlap of the sensitive ranges of individual energy bins. Hence, the spectral resolution and consequently the amount of spectral information gathered is reduced compared to ideal PCDs. Since the material separation capability strongly depends on the basis materials difference in absorption behavior among the energy bins, we expect the convergence rate of our algorithm to depend on the choice of energy bins.

The algorithm requires knowledge of the specific bin-spectra *𝒮* ^*b*^(*E*), that is, the part of the tube spectrum an energy bin is sensitive to, to correctly reconstruct material fractions; see (7). The specific spectra for arbitrary, realistic bins can be calculated via the detector response function Σ(*E*, *E*′); see (8). We acquired the response function with a resolution of 1 keV by simulating monoenergetic scans without phantom, with X-ray energies between 20.5 and 139.5 keV, using the SimSD simulation tool [30, 31]. The tool accounts for all relevant physical processes occurring in realistic PCDs. To keep the influence of pulse pileup on the detector response low (<1.5%), we chose a small X-ray flux of 4.8 · 10^6^ (1/s mm^2^) and used a clocked readout to discriminate pulses. The calculation of the response function assumes a virtual CdTe-detector with a bulk thickness of 1.6 mm, biased with a voltage of 1 kV, and the same (pixel) size as the detector described in Section 2.3.1. We sample the monoenergetic response functions with thresholds between 4 and 173 keV, again with a resolution of 1 keV. The detector response to each photon energy is approximated by averaging the response of 10k detector pixels. Figures 3(b)–3(d) show the sensitive range of two energy bins calculated from the detector response function for the chosen prefiltered 140 keV tube spectrum (cf. Section 2.3.1) for the investigated bin configurations. With the SimSD tool [30, 31] we also created realistic data sets from the cylindrical phantom for a 2-bin PCD, where realistic means that we consider pulse pileup, K-escape, charge sharing, and electronic noise.  Figure 2 shows the normalized, scaled total density ((*ρ*_tot_ − *ρ*_H_2_O_)/*ρ*_H_2_O_) · 1000 of the cylindrical phantom with image values centered at *C* = 0 with a window width of *W* = 100.

To investigate the dependence of convergence speed on the selected counter thresholds, we fixed the low energy counter at 20 keV and varied the high energy counter between 50 keV and 80 keV in steps of 15 keV. We created two data sets for each of the three energy bin configurations that only differ in their noise realization. This enables us to evaluate the image noise in difference images, avoiding systematic errors.

In comparison to ideal PCD data (cf. Section 2.3.1), a pileup correction is applied to the realistic raw data before reconstructing them with the proposed algorithm. The correction is based on a high-order polynomial that we fit to the mean true count rate plotted versus the respective mean count rate measured by the detector. We initialized the algorithm with the material fractions estimated from material separated FBP images. First, the FBP images are reconstructed from the simulated raw data after a combined water-BHC and pulse pileup correction had been applied. Next, the FBP images on Hounsfield scale are converted to attenuation values

(31) $$\begin{array}{c} \mu_{j}^{b}=\left(\;\frac{HU}{1000}-1\;\right)\mu_{j}^{b,H_{2}O} \end{array}$$

with

(32) $$\begin{array}{c} \mu_{j}^{b,H_{2}O}=\frac{\int_{E_{min}^{b}}^{E_{max}^{b}}\mu_{j}^{H_{2}O}\left(E\right)S^{b}\left(E\right)dE}{\int_{E_{min}^{b}}^{E_{max}^{b}}S^{b}\left(E\right)dE}. \end{array}$$

Finally, the initial material fractions **f** ^(0)^ are calculated from the attenuation maps *μ*_*j*_^*b*^ via

(33) $$\begin{array}{c} f^{\left(0\right)}=M^{-1}\mu , \end{array}$$

with **f** ^(0)^ ≡ {*f*_*j*_^*k*,(0)^} and ***μ*** ≡ {*μ*_*j*_^*b*^}. The matrix *ℳ* ≡ *μ* ^*kb*^ contains the attenuation values of the *K* pure basis materials for the respective bin-spectrum *𝒮* ^*b*^(*E*). *μ* ^*kb*^ can be calculated using the tabulated attenuation values from [26]

(34) $$\begin{array}{c} \mu^{kb}=\frac{\int_{E_{min}^{b}}^{E_{max}^{b}}\mu^{k}\left(E\right)S^{b}\left(E\right)dE}{\int_{E_{min}^{b}}^{E_{max}^{b}}S^{b}\left(E\right)dE}. \end{array}$$

## 3. Results

### 3.1. Accuracy Analysis with Ideal PCD Data

**Figure 4:**
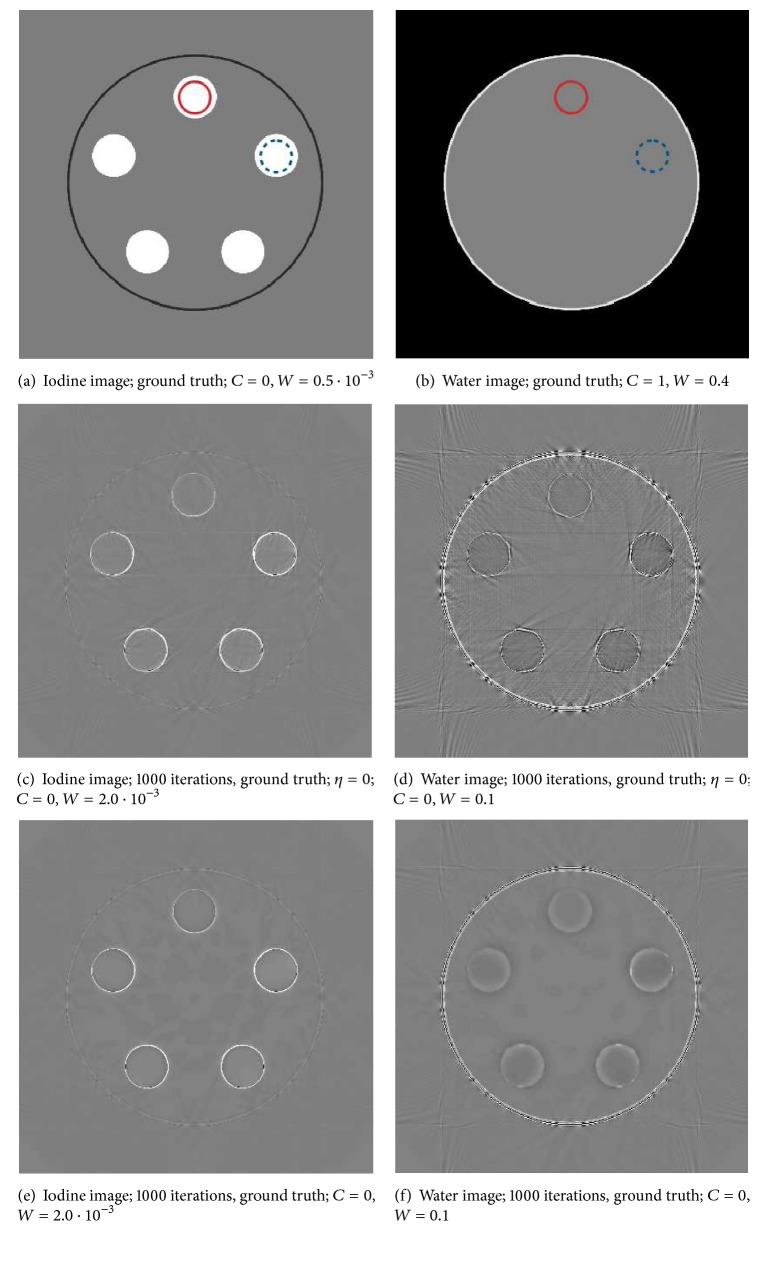
Reconstructed material images from data of an ideal PCD with the proposed algorithm with an energy resolution of 5 keV, after 1000 iterations and a subtraction of the respective ground truth image.

We compared the results of two different reconstructions of the same raw data sets. In the first case we reconstructed the material images from the ideal PCD data without any regularization; that is, **η** = 0. For the second case we employed regularization with *η* ^*k*^ · *γ* ^*k*^ = 1 · 10^−5^. Parameters *γ* ^*k*^ were selected depending on the considered material *k* by taking into account the image noise contained in the respective initial material images reconstructed from* realistic* data. After 1000 iterations the material fractions as well as their uncertainties were evaluated for both cases within the two ROIs that are depicted in Figures 4(a) and 4(b). ROI_1_ is indicated by a dashed circle located within the highest iodine contrast cylinder and at the respective position in the water image. ROI_2_ is indicated by a solid circle located at the lowest iodine contrast cylinder and at the respective position in the water image. The results of the ROI analysis are summarized in Table 1. The table also lists the mean deviations from the ground truth Δ*f* ^*∗*^ = |*f* ^*∗*,(0)^ − *f* ^*∗*,(1000)^| within the ROIs.  Figure 4 shows the true material images for iodine (a) and water (b), as well as the difference between the respective images and the ground truth after 1000 iterations for both investigated cases ((c)–(f)).

### 3.2. Dependence of Convergence Speed on Energy Bin Selection

**Figure 5:**
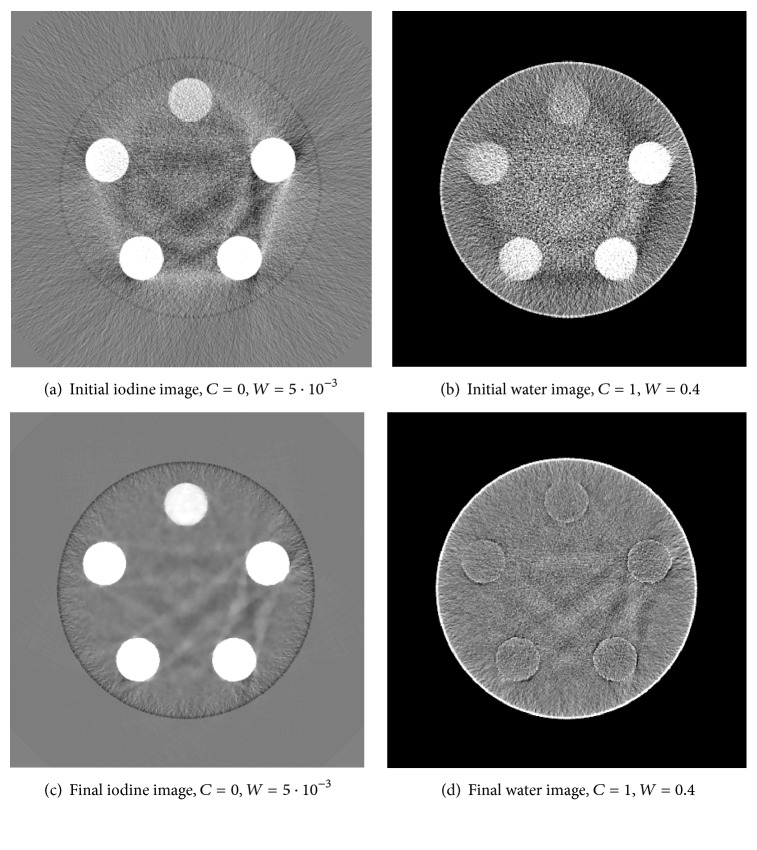
Initial ((a), (b)) and final ((c), (d)) material images from data set of a realistic PCD with the proposed algorithm after 1000 iterations. The selected regularization parameters were *γ* ^iodine^ = 8.6 · 10^−5^, *γ* ^H_2_O^ = 1.4 · 10^−2^, and *η* ^*∗*^ · *γ* ^*∗*^ = 1 · 10^−5^.

The initial material images as well as the final material images after 1000 iterations are exemplarily shown in Figure 5 for the data set featuring the (20–65 keV, 65–140 keV) bin configuration. Compared to the ground truth images shown in Figures 4(a) and 4(b) significant beam-hardening artifacts are visible between the iodine contrast probes. They are caused by the FBP reconstruction based on the monoenergetic version of Lambert-Beer's law. Due to these artifacts, the iodine contrast is considerably underestimated and images of the iodine contrast probes also show up in the initial water material image.

Similarly to the ideal material images in Section 2.3.1, all material images have been evaluated within identically positioned ROIs. The image noise, that is, the standard deviation of the ROI data, is not estimated directly in the reconstructed images. Instead, the images reconstructed from the two data sets that only differ in their noise realization are subtracted and the image noise is evaluated within the resulting difference image. Eventually, the measured image noise is scaled by a factor of $1/\sqrt{2}$ to account for Gaussian error propagation.

**Figure 6:**
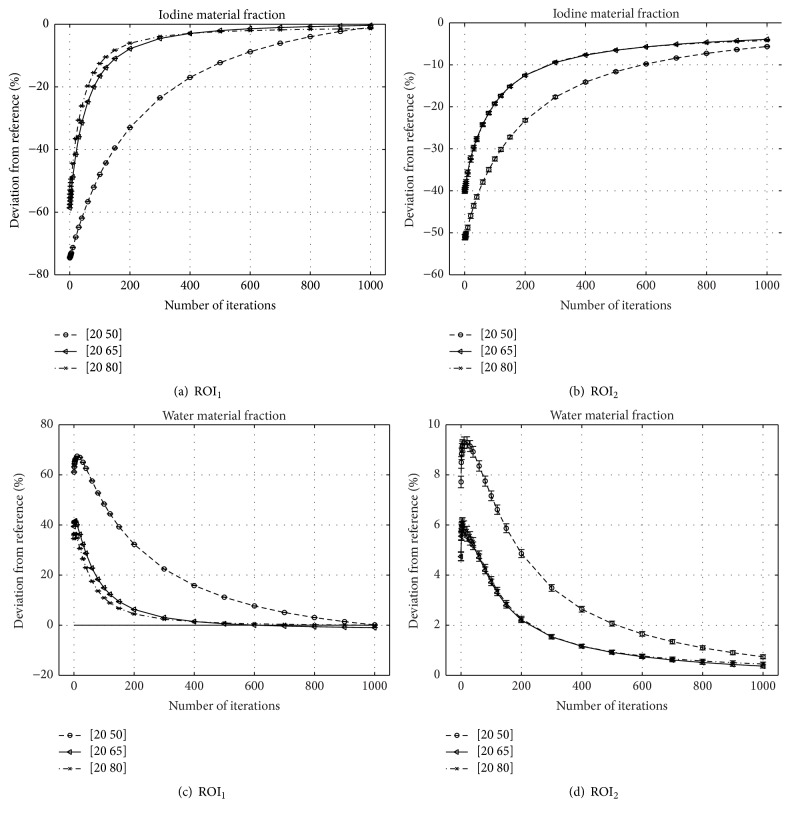
Normalized deviation of the iodine ((a), (b)) and water ((c), (d)) fraction from the ground truth (Δ*f* ^iodine∣water^/*f* ^GT^), evaluated within the ROIs shown in Figures 4(a) and 4(b), respectively.

The deviation of the measured material fraction *f* ^*∗*,(1000)^ from the ground truth as well as the respective image noise is plotted against iteration number in Figure 6 for both basis materials and the various bin configurations.

## 4. Discussion

### 4.1. Accuracy Analysis with Ideal PCD Data

Considering the results of the reconstruction with deactivated regularization (see Figures 4(c) and 4(d)), the chosen internal energy resolution of 5 keV seems sufficient, since the deviations Δ*f* ^*∗*^ from the ground truth are very small after 1000 iterations. The final images from nonregularized reconstruction mainly exhibit moiré pattern residuals that are typical discretization artifacts from the forward- and back-projectors. Those artifacts are much more prominent than the high-frequency, random noise residuals expected from a statistical reconstruction algorithm. So we conclude that the standard deviations *σ*_*f*^*∗*^_ calculated from the ROI data are a rough measure of discretization artifacts. The standard deviation is of equal magnitude as the deviations Δ*f* ^*∗*^, which are mainly caused by the limited internal energy resolution. This confirms that for the utilized projection operator an internal energy resolution of 5 keV is adequate.

If regularization is employed, standard deviation in both material images is reduced, especially in the contrast probes within the iodine image. In return though, the precision of the material fraction estimation is reduced slightly for data within ROI_1_, that is, the probe with the highest iodine contrast. We suppose that this loss in precision comes from the way regularization is applied to the material images. Although the material images are linked by measured spectral data sets *l*_*i*_(*E*, **f**) = ∑_*k*=1_^*K*^∑_*j*=1_^*P*^ *a*_*ij*_ *f*_*j*_^*k*^ *μ* ^*k*^(*E*), regularization is applied to each material image individually. If the regularization is not perfectly edge-preserving this leads to a bias, affecting the precision of the material image estimate. Suppose that by regularization of the iodine material image the edges of a contrast probe are smoothed out as illustrated in Figure 8(a). The requirement

(35) $$\begin{array}{c} f_{j}^{iodine}\mu^{iodine}\left(\;E\;\right)+f_{j}^{H_{2}O}\mu^{H_{2}O}\left(\;E\;\right)=\text{const}. \end{array}$$

and by far stronger attenuation coefficient of iodine in the considered energy window inevitably cause the smoothed out edge to reappear in the water image with increased amplitude; see Figure 8(b). The regularization of the water image assures that the imprinted edges from the iodine image get smoothed and blurred and in turn also influence the iodine, albeit to a much lesser extent. Thus, accumulated over the number of iterations this effect slightly influences the mean contrast measured within the ROIs in both images. The more prominent the contrast edge, the stronger the resulting deviation caused by regularization (cf. Table 1). A strictly edge-preserving prior like the Huber prior might reduce this issue but was not tested, since it does not fulfill the convergence criteria according to [16] and a divergence of the algorithm seemed likely.

### 4.2. Dependence of Convergence Speed on Energy Bin Selection

Generally, the choice of the second derivative of *h*_*i*_^*b*^(*E*, *l*_*i*_) seems to be valid, since in all investigated cases the algorithm converged. As conjectured, it proves true that the convergence speed depends on the selected thresholds. While for thresholds located at 20∣65 keV and 20∣80 keV the algorithm shows a similar convergence rate (cf. Figure 6), it converges noticeably slower for the threshold combination 20∣50 keV. On the one hand, this is due to a reduced separation between the two bin-spectra *𝒮* ^*b*^(*E*); see Figure 3(b). On the other hand, it is a consequence of comparably worse initial images which are affected by the large overlap of the bin-spectra as well. Therefore, not only is an increase of the number of thresholds in a PCD necessary to allow a separation of more than two basis materials, but the respective data sets also have to be spectrally well separated. In addition, the absorption behavior of the basis materials must be sufficiently distinct. For instance, a separation of water and fat will hardly converge with the proposed algorithm due to the similar absorption behavior of these materials within the energy range of clinical CT.

Addressing the dark shades between the contrast probes, caused by beam-hardening of the polychromatic X-ray tube spectrum, those artifacts are noticeably reduced after 1000 steps of iteration.

Remarkably, the image noise measured in ROI_1_ within the highest contrast rod of the iodine image is increasing beyond the respective image noise within the FBP starting image. This happens since within the first iterations mainly the mean iodine contrast is scaled to match the respective sinogram data. Scaling all image values by a certain factor affects the standard deviation as well. Since the initial estimate of the highest iodine contrast is not very accurate and regularization primarily limits high-frequency noise from being added to the material images, it cannot prevent an initial increase of image noise in this case. For the smallest contrast, the initial image represents a decent estimate of the actual iodine fraction and changes made by the algorithm within the first few iterations are less severe, so an increase of image noise can be prevented by regularization.

Summarizing the results of Figure 6, the algorithm performs well in correcting beam-hardening artifacts and quantitatively determining the material fractions for a two-bin PCD, if the material basis is chosen properly.

**Figure 7:**
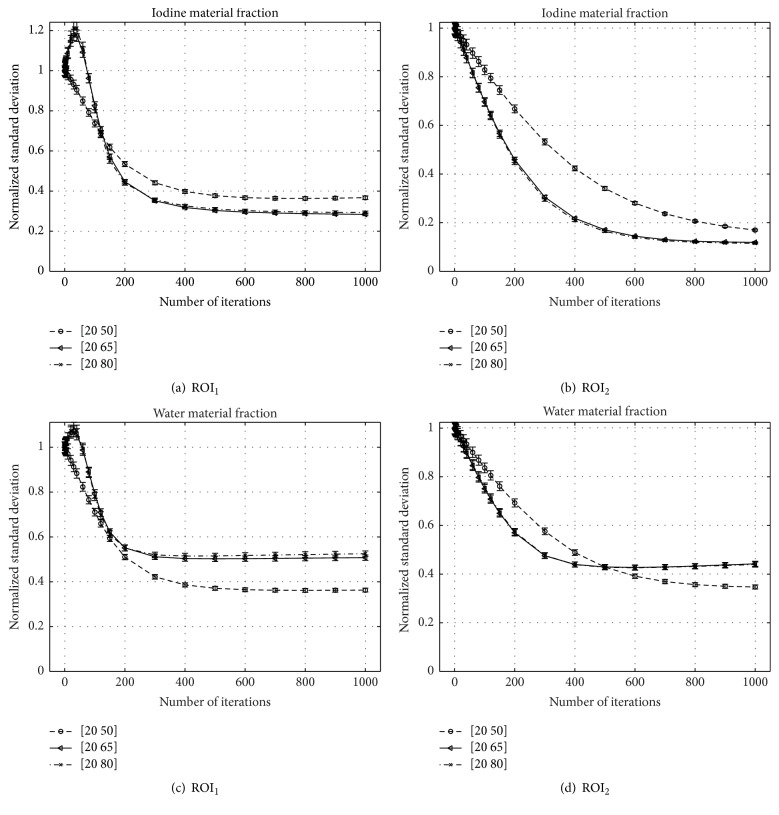
Normalized standard deviation, evaluated within the ROIs shown in Figures 4(a) and 4(b), respectively. The standard deviation is normalized to the standard deviation measured in the initial iodine or water image for the respective threshold combination.

**Figure 8:**
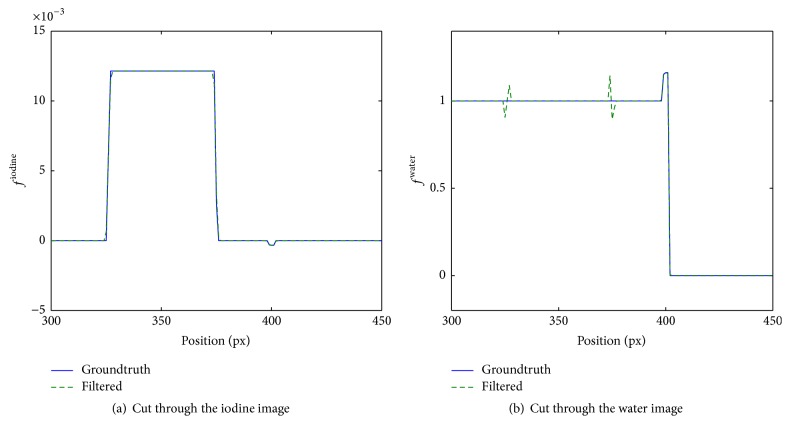
Illustration of the impression of edges from the iodine image onto the water image at *E* = 65 keV (cf. (35)). Shown are cuts through the material images crossing the highest contrast cylinder of the phantom shown in Figure 2.

With the utilized regularization function and parameters a significant reduction of image noise in both material images was achievable; see Figure 7.

## 5. Conclusions

An iterative statistical reconstruction algorithm has been introduced that successively approximates the negative log-likelihood function by paraboloidal surrogate functions. With the proposed algorithm a direct reconstruction of a set of material images is possible from energy-resolved sinogram data. Since the algorithm considers the polychromatic nature of X-rays generated by typical clinical X-ray sources the algorithm includes an implicit beam-hardening correction for the selected basis materials. Apart from that the algorithm has been tailored for reconstruction of material images from data measured with photon-counting detectors by taking into account correlations introduced between energy bin data sets in the detection process via the detector response function. It was shown that an internal energy resolution of 5 keV is sufficient to yield quantitative results if a proper material basis is selected. Compared to reconstruction algorithms utilized on current commercial scanners the proposed algorithm converges rather slowly. This might make an immediate implementation in those scanners unlikely, despite the possibility for parallel computation.

## Figures and Tables

**Table 1:** Results of the accuracy analysis of the algorithm with and w/o regularization (regul.). The material fractions *f* ^*∗*,(1000)^ were measured after 1000 iterations in the respective material images within ROIs located as indicated in Figures 4(a) and 4(b). The standard deviation of the ROI data *σ*_*f*^*∗*^_ measures the fluctuation induced by the forward- and back-projector. The values of Δ*f* ^*∗*^ quantify the offset between the ground truth *f* ^*∗*,(0)^ and the material fraction after 1000 iterations.

		W/o regul.	With regul.
ROI_1_	*f* ^iodine,(1000)^	0.01220	0.01221
	Δ*f* ^iodine^	0.54 · 10^−4^	0.66 · 10^−4^
	*σ*_*f*^iodine^_	0.32 · 10^−4^	0.18 · 10^−4^
	*f* ^H_2_O,(1000)^	0.99818	0.99770
	Δ*f* ^H_2_O^	0.18 · 10^−2^	0.23 · 10^−2^
	*σ*_*f*^H_2_O^_	0.19 · 10^−2^	0.18 · 10^−2^

ROI_2_	*f* ^iodine,(1000)^	0.00245	0.00245
	Δ*f* ^iodine^	0.17 · 10^−4^	0.18 · 10^−4^
	*σ*_*f*^iodine^_	0.14 · 10^−4^	0.08 · 10^−4^
	*f* ^H_2_O,(1000)^	0.99940	0.99965
	Δ*f* ^H_2_O^	0.60 · 10^−3^	0.35 · 10^−3^
	*σ*_*f*^H_2_O^_	0.15 · 10^−2^	0.12 · 10^−2^

## References

[1] Flohr T. G., McCollough C. H., Bruder H. (2006). First performance evaluation of a dual-source ct (dsct) system. *European Radiology*.

[2] Kalender W. A., Perman W. H., Vetter J. R., Klotz E. (1986). Evaluation of a prototype dual-energy computed tomographic apparatus. I. Phantom studies. *Medical Physics*.

[3] Kelcz F., Joseph P. M., Hilal S. K. (1979). Noise considerations in dual energy CT scanning. *Medical Physics*.

[4] Carmi R., Naveh G., Altman A. Material separation with dual-layer CT.

[5] Ballabriga R., Campbell M., Heijne E. H. M., Llopart X., Tlustos L. The medipix3 prototype, a pixel readout chip working in single photon counting mode with improved spectrometric performance.

[6] Kappler S., Henning A., Krauss B. Multi-energy performance of a research prototype CT scanner with small-pixel counting detector.

[7] Weidinger T., Buzug T. M., Flohr T. Investigation of ultra low-dose scans in the context of quantum-counting clinical CT.

[8] Kappler S., Niederlöhner D., Stierstorfer K., Flohr T. Contrast-enhancement, image noise, and dual-energy simulations for quantum-counting clinical CT.

[9] Kappler S., Glasser F., Janssen S., Kraft E., Reinwand M. A research prototype system for quantum-counting clinical CT.

[10] Szeles C., Soldner S. A., Vydrin S., Graves J., Bale D. S. (2008). CdZnTe semiconductor detectors for spectroscopic X-ray imaging. *IEEE Transactions on Nuclear Science*.

[11] Johnson T. R. C., Fink C., Schonberg S. O., Reiser M. F. (2011). *Dual Energy CT in Clinical Practice*.

[12] Desai M. A., Peterson J. J., Garner H. W., Kransdorf M. J. (2011). Clinical utility of dual-energy CT for evaluation of tophaceous gout. *Radiographics*.

[13] Thomas C., Korn A., Krauss B. (2010). Automatic bone and plaque removal using dual energy CT for head and neck angiography: feasibility and initial performance evaluation. *European Journal of Radiology*.

[14] Marshall W. H., Alvarez R. E., Macovski A. (1981). Initial results with prereconstruction dual-energy computed tomography (PREDECT). *Radiology*.

[15] Buzug T. M. (2008). *Computed Tomography*.

[16] De Pierro A. R. (1995). Modified expectation maximization algorithm for penalized likelihood estimation in emission tomography. *IEEE Transactions on Medical Imaging*.

[17] Fessler J. A., Elbakri I. A., Sukovic P., Clinthorne N. H. Maximum-likelihood dual-energy tomographic image reconstruction.

[18] Erdoğan H., Fessler J. A. (1999). Monotonic algorithms for transmission tomography. *IEEE Transactions on Medical Imaging*.

[19] Elbakri I. A., Fessler J. A. (2002). Statistical image reconstruction for polyenergetic X-ray computed tomography. *IEEE Transactions on Medical Imaging*.

[20] Elbakri I. A., Fessler J. A. (2003). Segmentation-free statistical image reconstruction for polyenergetic X-ray computed tomography with experimental validation. *Physics in Medicine and Biology*.

[21] la Riviere P. J., Vargas P. Penalized-likelihood sinogram decomposition for dual-energy computed tomography.

[22] Green P. J. (1990). Bayesian reconstructions from emission tomography data using a modified EM algorithm. *IEEE Transactions on Medical Imaging*.

[23] Huber P. J. (1964). Robust estimation of a location parameter. *Annals of Mathematical Statistics*.

[24] Sutcliffe J. F. (1996). A review of in vivo experimental methods to determine the composition of the human body. *Physics in Medicine and Biology*.

[25] Ellis K. J. (2000). Human body composition: in vivo methods. *Physiological Reviews*.

[26] Cullen D. E., Hubbell J. H., Kissel L. (1997). Epdl97: the evaluated photon data library, '97 version.

[27] De Pierro A. R. (1993). On the relation between the ISRA and the EM algorithm for positron emission tomography. *IEEE Transactions on Medical Imaging*.

[28] Stierstorfer K. (2000). Drasim—deterministic radiological simulation. *Technical Report, Internal Report*.

[29] Kuchling H. (2004). *Taschenbuch der Physik*.

[30] Balda M., Niederlöhner D., Kreisler B., Durst J., Heismann B. Lookup table-based simulation of directly-converting counting X-ray detectors for computed tomography.

[31] Balda M. (2011). *Quantitative computed tomography [Diss.]*.

