# Corrigendum to “Polychromatic Iterative Statistical Material Image Reconstruction for Photon-Counting Computed Tomography”

**DOI:** 10.1155/2018/5932653

**Published:** 2018-08-09

**Authors:** Thomas Weidinger, Thorsten M. Buzug, Thomas G. Flohr, Steffen Kappler, Karl Stierstorfer

**Affiliations:** ^1^Institute of Medical Engineering, University of Lübeck, Ratzeburger Allee 160, 23538 Lübeck, Germany; ^2^Siemens AG, Healthcare Sector, Imaging & Therapy Division, Siemensstraße 1, 91301 Forchheim, Germany

In the article titled “Polychromatic Iterative Statistical Material Image Reconstruction for Photon-Counting Computed Tomography” [1], there were errors in equations (13) and (28) in addition to some errors in the text. This should be corrected as follows:

(1) Equation (13) should be corrected to

(13) $$\begin{array}{c} \beta_{i}^{b,\left(n\right)}\left(\;E\;\right)=\frac{{\overset{-}{N}}_{i}^{b,\left(n\right)}\left(f^{\left(n\right)}\right)}{t_{i}\left(E,f^{\left(n\right)}\right)}. \end{array}$$

(2) The text between equations (14) and (15) should be corrected to

(14) $$\begin{array}{c} {\overset{-}{N}}_{i}^{b}\left(\;f\;\right)=\int \frac{N_{i,0}^{b}\left(E\right)}{\beta_{i}^{b,\left(n\right)}\left(E,f^{\left(n\right)}\right)}t_{i}\left(\;E,f\;\right)\beta_{i}^{b,\left(n\right)}\left(\;E,f^{\left(n\right)}\;\right)dE. \end{array}$$

Since *h*_*i*_^*b*^ is a convex function, it holds that

(15) $$\begin{array}{c} \int \frac{N_{i,0}^{b}\left(E\right)}{\beta_{i}^{b,\left(n\right)}\left(E,f^{\left(n\right)}\right)}dE=1. \end{array}$$

(3) Equation (28) should be corrected to

(28) ∂2Q3∂fjk∂fjm=∑b=1B∑i=1N∫Ni,0bEβib,nαijaij2μkEμmEN−ib,ndE=∑b=1B∑i=1NaijNibN−ib∑j=1Paij·∫Ni,0bEμkEμmEexp⁡−lindE.

(4) In the “Summary of the Algorithm” subsection, step 3 should be corrected to

(3) Additionally, calculate

(30) $$\begin{array}{c} \int N_{i,0}^{b}\left(\;E\;\right)\mu^{k}\left(\;E\;\right)\exp\left(\;-\overset{P}{\underset{j=1}{\sum }}a_{ij}\mu_{j}\left(\;E\;\right)\;\right)dE, \end{array}$$

which equates to $-\left(1/a_{ij}\right)\left(\partial {\overset{-}{N}}_{i}^{b,\left(n\right)}/\partial f_{j}^{k}\right)$.
